# Comparing Lightweight Models for Physician-Consensus Pharyngitis Classification from Primary-Care Smartphone Images

**DOI:** 10.3390/diagnostics16213552

**Published:** 2026-10-28

**Authors:** Adnan Miski, Mazen A. Ismail, Anas A. Makki

**Affiliations:** 1 Department of Industrial Engineering, Faculty of Engineering—Rabigh, King Abdulaziz University, Jeddah 21589, Saudi Arabia; nhmakki@kau.edu.sa; 2 Department of Medical Education, Faculty of Medicine, King Abdulaziz University, Jeddah 21589, Saudi Arabia; mabismail@kau.edu.sa

**Keywords:** pharyngitis, physician consensus, deep learning, smartphone imaging, lightweight neural networks, acquisition-device association

## Abstract

**Background/Objectives:** Smartphone-acquired throat images could support accessible pharyngitis assessment, but the performance and computational efficiency of lightweight models on such images require further evaluation. This study compared five lightweight models for predicting physician-consensus bacterial versus nonbacterial labels from smartphone throat images. **Methods:** We analyzed PGUPharyngitis, a public dataset of smartphone throat images collected in general practice. The final cohort comprised 635 patients, of whom 105 (16.5%) had bacterial physician-consensus labels. The reference outcome was physician consensus based on images and clinical information rather than microbiological testing. Five ImageNet-pretrained lightweight classifiers—four convolutional neural networks (CNNs; MobileNetV3-Small, EfficientNet-B0, ShuffleNetV2, and YOLO26n-cls) and one CNN–Transformer hybrid (MobileViT-XS)—were fine-tuned using patient-level five-fold cross-validation. Performance was assessed using area under the precision–recall curve (PR-AUC) and area under the receiver operating characteristic curve (AUROC), alongside computational efficiency. Device-only and clinical-variable logistic-regression models provided non-image comparisons. **Results:** Mean PR-AUC across folds ranged from 0.317 to 0.372 and AUROC from 0.663 to 0.708, with no clearly superior architecture. Bacterial-label prevalence differed by acquisition device (Samsung 9.2% versus Xiaomi 26.9%); the device-only and clinical-variable comparators achieved AUROCs of 0.650 and 0.598, respectively. At validation-selected high-sensitivity thresholds, the models flagged 65–78% of patients, with positive predictive values of only 19–22%. All models contained fewer than five million parameters, making them compact candidates for future evaluation on resource-constrained devices. **Conclusions:** Lightweight models showed moderate performance against physician-consensus labels. However, acquisition device was associated with the reference labels, image-model AUROCs were not clearly distinguishable from that of the device-only comparator, and device-related confounding cannot be excluded. Because performance was measured against these labels and positive predictive values were low, the findings do not establish diagnostic accuracy or clinical applicability, which require validation against microbiological reference standards in independent clinical cohorts.

## 1. Introduction

Acute pharyngitis is common in primary care, but overlapping symptoms make bacterial and viral causes difficult to distinguish [1]. Current guidance uses clinical scores to identify patients who may need testing for group A Streptococcus (GAS), rather than to establish the diagnosis alone [2]. Rapid antigen tests provide point-of-care evidence; a pediatric Cochrane review reported sensitivity of 85.6% and specificity of 95.4% against throat culture [3]. Frequent antibiotic prescribing for sore throat motivates better assessment while avoiding unnecessary treatment [4,5].

Smartphone throat photographs could support remote assessment, and lightweight networks reduce the memory and computing requirements of mobile systems [6,7]. Prior image-based studies have addressed heterogeneous targets and modalities. Askarian et al. classified strep-throat cases versus healthy controls using smartphone images, color features, and a custom optical attachment [8]. Other studies distinguished severe or exudative pharyngitis from normal or non-pharyngitis images [6,9,10], while Swathi and Regunathan evaluated a four-class feature-fusion pipeline for pharyngitis, tonsillitis, oral cancer, and healthy images [11]. Hoffer et al. studied smartphone-connected infrared thermography in children; thermal imaging alone did not distinguish streptococcal from viral pharyngitis, although combining it with the McIsaac score improved prediction [12]. Asokan et al. also examined computational efficiency in a throat-image pipeline [13]. Because these studies differ in target condition, imaging modality, inputs, and reference standards, their reported performance is not directly comparable with classification of physician-consensus bacterial versus nonbacterial labels from smartphone throat images.

PGUPharyngitis is a publicly available dataset of smartphone-acquired throat images and accompanying clinical information collected in general practice, with multiple physician assessments supporting bacterial-versus-nonbacterial classification [14]. Although microbiological reference results are unavailable, the physician-consensus outcome provides a practical operational reference for comparing lightweight architectures under a shared evaluation protocol. The dataset’s public availability and released physician assessments also enable transparent and reproducible benchmarking.

Previous studies using the dataset have provided initial benchmarks for image-based classification. The original image-model baselines achieved areas under the receiver operating characteristic curve (AUROCs) of approximately 0.553–0.645 across the full dataset [14]. In a subsequent analysis, Rahman excluded 102 ambiguous cases and reported image-only AUROCs of 0.673 and 0.705, but sensitivities for bacterial-labeled cases remained low at 10.4% and 8.3%, respectively [15]. Beyond PGUPharyngitis, Hamdy et al. reported an AUROC of 0.79 for StrepApp, which combines throat images with clinical data, in a prospective pediatric cohort undergoing GAS testing [16]. Rwebembera et al. evaluated artificial intelligence (AI) analysis of smartphone-acquired throat videos against culture in a prospective pilot study of 82 Ugandan children, including 12 with culture-confirmed GAS [17]. These studies addressed diagnostic performance against microbiological testing, whereas the present study evaluates the comparative performance and computational efficiency of lightweight models using physician-consensus labels.

The contrast between moderate AUROCs and low sensitivities in earlier work highlights the need to evaluate lightweight models beyond overall classification performance. Their practical value depends on computational cost and the balance between detecting bacterial-labeled cases and incorrectly flagging nonbacterial-labeled cases. Precision–recall analysis is particularly useful when the positive class is a minority [18]. Acquisition conditions also warrant examination because medical-image models can exploit device-related signals associated with the reference labels [19,20]. Evaluating these aspects under a shared protocol would help determine whether compact models merit further development for smartphone-based primary-care decision support and identify the acquisition and performance limitations that must be addressed before evaluating them on other camera-equipped portable or wearable devices.

Accordingly, the primary aim was to compare five lightweight image-classification architectures under a shared patient-level five-fold cross-validation protocol using physician-consensus labels. The study evaluated classification performance, high-sensitivity operating characteristics, computational efficiency, and device-associated variation, with separate device-only and clinical-variable models providing additional context. Although evaluated only on smartphone-acquired images, these compact architectures may also be relevant to other camera-equipped portable or wearable platforms, provided they are validated on each device under the intended acquisition conditions.

## 2. Materials and Methods

### 2.1. Study Design and Dataset

This secondary analysis used PGUPharyngitis [14], a public dataset collected from patients with common-cold-related symptoms attending general practitioners in two Iranian regions between October 2023 and May 2024. The source investigators retained 742 of 860 images after quality review, obtained written informed consent, and reported ethics approval IR.BPUMS.REC.1403.282. No new participants or clinical data were collected. Reporting was guided by the TRIPOD+AI statement for clinical prediction-model studies [21]; a completed reporting checklist accompanies the manuscript.

Labels were reconstructed by a prespecified strict majority of released physician votes. We excluded 100 ties, one majority-labeled case without a mapped image, and all six records from three byte-identical cross-patient image pairs whose patient attribution could not be resolved. The final cohort comprised 635 patients: 105 bacterial (16.5%) and 530 nonbacterial (83.5%). This distribution reflects the reconstructed study cohort after exclusions and should not be interpreted as the population prevalence of bacterial pharyngitis. Throughout the paper, “bacterial” and “nonbacterial” refer to physician-consensus reference labels rather than etiologic diagnoses confirmed by microbiological testing, such as throat culture, GAS rapid antigen detection testing, or molecular assays. Accordingly, the reported classification metrics evaluate performance against these physician-consensus labels and do not establish diagnostic accuracy for microbiologically confirmed bacterial infection. The source describes annotation using images and symptoms, with one physician examining the throat directly and others reviewing images [14]. The reference labels therefore share information with the evaluated inputs, creating potential incorporation bias. The source nonbacterial category includes viral, allergic, and normal presentations.

An audit of the released files against the source report identified several discrepancies: 741 readable throat images could be mapped to the 742 spreadsheet records; the released files yielded 234 reconstructed unanimous cases, compared with 182 full-consensus cases reported in the source article; and the released spreadsheet contained a mean of 4.98 physician votes per patient, compared with approximately six reported. Device metadata also differed from the reported handset description [14,22]. We therefore reconstructed the cohort from the released files; Table S1 details the audit.

**Figure 1:**
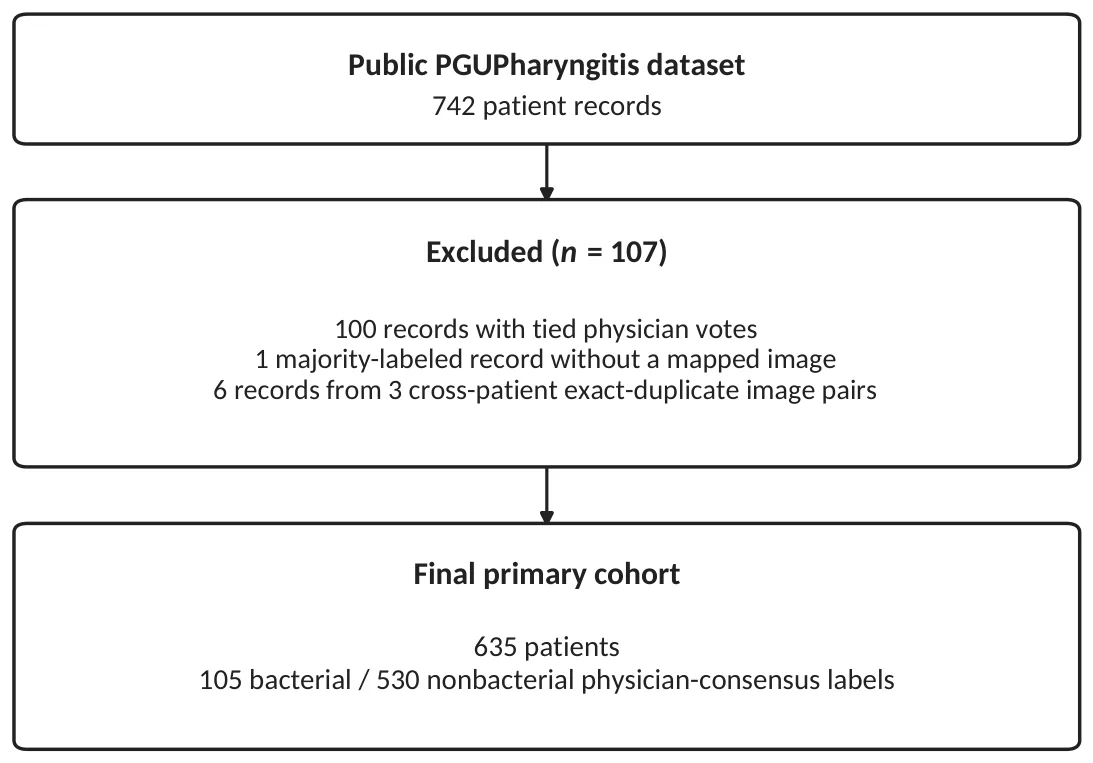
Selection of the primary analysis cohort and reasons for exclusion.

**Figure 2:**
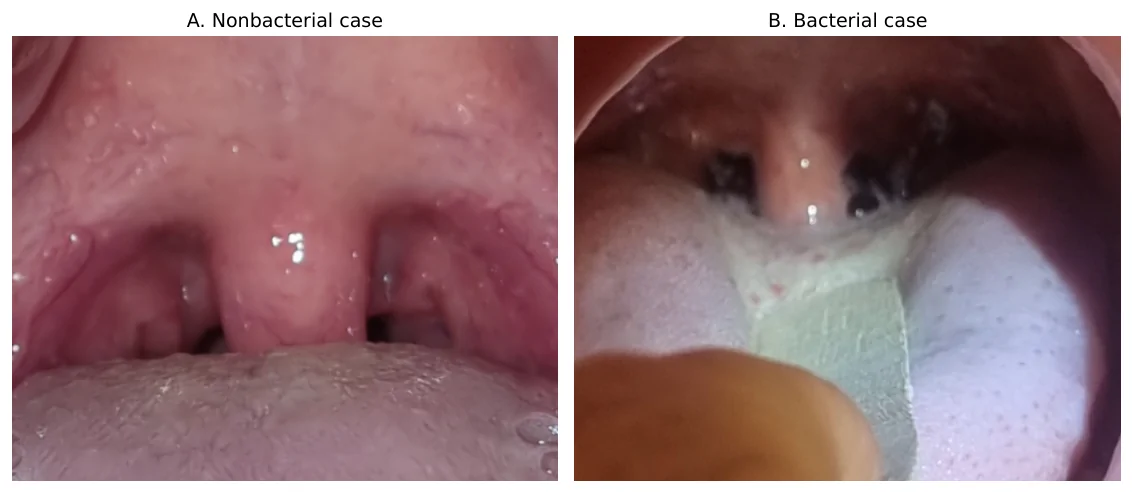
Representative throat images from the PGUPharyngitis dataset deposit [14]: (**A**) a patient unanimously labeled nonbacterial and (**B**) a patient unanimously labeled bacterial by the participating physicians. The Figshare deposit is licensed under the Creative Commons Attribution 4.0 International license; the source images were resized and arranged for presentation.

Acquisition device was identified from Exchangeable Image File Format (EXIF) metadata as Samsung or Xiaomi and was unavailable for 54 patients. Table 1 and Table 2 describe the cohort; Figure 1 and Figure 2 show selection and examples. Symptom percentages in Table 2 represent patient-level prevalence and therefore use a different denominator from the percentages reported in the source publication.

### 2.2. Models and Image Preprocessing

Prediction of physician-consensus bacterial versus nonbacterial labels from throat images was formulated as a supervised binary task, with the bacterial-labeled category defined as the positive class. All image classifiers were deep neural networks; logistic regression was used only for the non-image device-only and clinical-variable comparators (Section 2.4).

Four convolutional neural network (CNN)-based architectures were evaluated: MobileNetV3-Small [7], EfficientNet-B0 [23], ShuffleNetV2 ×1.0 [24], and YOLO26n-cls [25]. CNNs learn hierarchical spatial features, such as edges, textures, and shapes, directly from images. MobileViT-XS [26] was the only CNN–Transformer hybrid; it combines convolutional layers for local feature extraction with transformer self-attention for modeling broader spatial relationships. MobileNetV3-Small served as the baseline architecture because it had previously been evaluated on PGUPharyngitis [14]. All five models used ImageNet-pretrained weights and were fine-tuned end-to-end. Their architecture families and trainable parameter counts are summarized in Table 3.

Images were resized to 224×224 pixels. Training-only augmentation comprised horizontal flipping, small rotations and translations, mild scaling, and limited brightness, contrast, and saturation variation. Validation and test images were not augmented. After conversion to tensors, MobileNetV3-Small, EfficientNet-B0, and ShuffleNetV2 used ImageNet channel normalization with means of 0.485, 0.456, and 0.406 and standard deviations of 0.229, 0.224, and 0.225. MobileViT-XS and YOLO26n-cls used zero-to-one pixel scaling without additional standardization. The corresponding model-specific preprocessing was applied consistently to the training, validation, and test images.

### 2.3. Cross-Validation and Training

**Figure 3:**
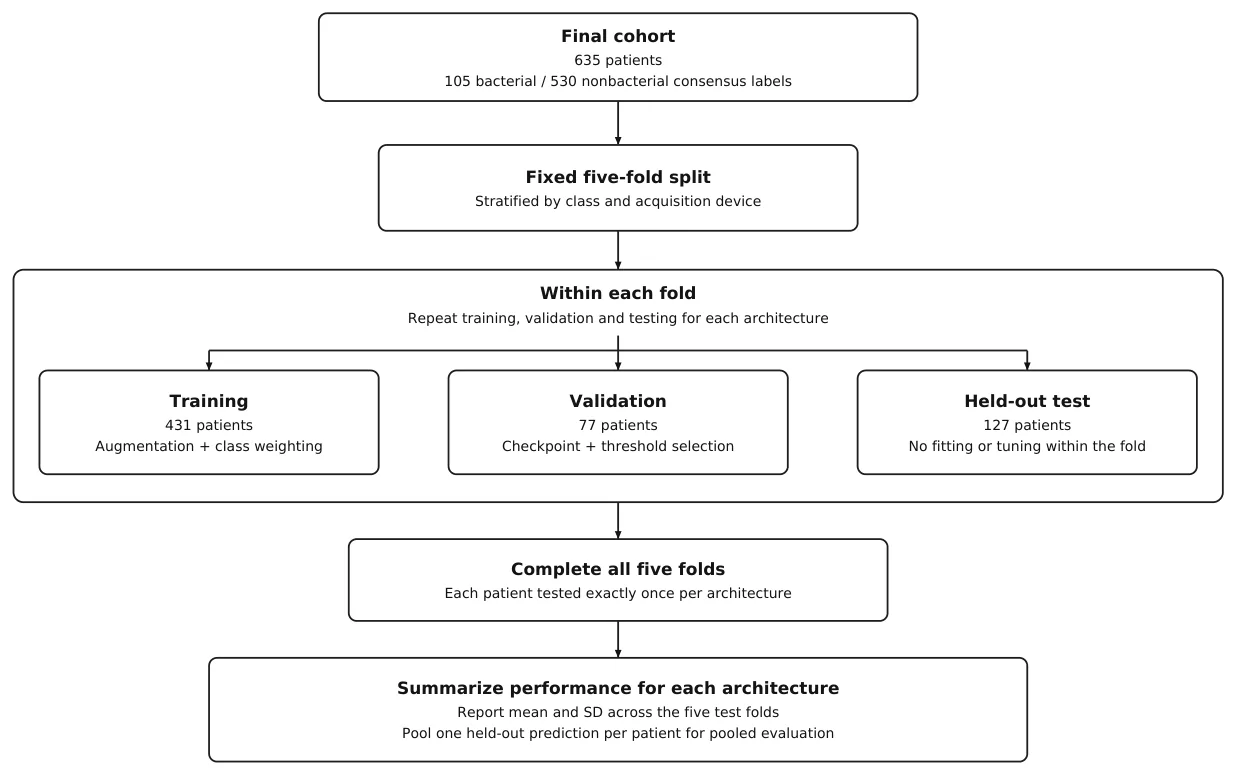
Five-fold cross-validation workflow.

The primary evaluation used fixed five-fold patient-level cross-validation on all 635 patients, stratified jointly by class and acquisition-device stratum. Each fold contained 431 training, 77 validation, and 127 held-out test patients, with every patient tested exactly once (Table 4; Figure 3). Exact duplicates were removed before splitting. Class weights and augmentation used training data only; early stopping, checkpoint selection, and high-sensitivity threshold selection used validation data only; held-out test patients were used only for final scoring.

During a preliminary 70/15/15 pipeline-development run on the same cohort, an initial learning rate of $3\times 10^{-4}$ produced rapid training–validation divergence, with validation-loss minima at epoch 1 for multiple fully fine-tuned architectures. The learning rate was therefore reduced globally to $1\times 10^{-4}$ before the primary cross-validation, with no other training hyperparameters changed in response to the pilot. Only the subsequent five-fold cross-validation results are reported.

The final shared protocol used AdamW (learning rate $1\times 10^{-4}$; weight decay $1\times 10^{-4}$), batch size 32, at most 60 epochs, inverse-frequency weighted cross-entropy, cosine learning-rate annealing, and early stopping with patience 12. No architecture-specific tuning was performed; complete training and augmentation settings are provided in Table S1.

### 2.4. Performance Measures and Statistical Analysis

The area under the precision–recall curve (PR-AUC) and AUROC were the prespecified primary measures; PR-AUC addresses performance for the minority bacterial-labeled class [18]. Throughout this study, PR-AUC denotes average precision computed using sklearn.metrics.average_precision_score in scikit-learn 1.6.1, rather than trapezoidal integration of the precision–recall curve. Secondary measures were sensitivity and specificity, with F1, macro-F1, and balanced accuracy retained as supplementary summaries. We evaluated a score threshold of 0.50 and an exploratory high-sensitivity threshold. For each fold, the latter maximized validation specificity subject to sensitivity of at least 90%, then was applied unchanged to the test patients. Each validation set contained only 12–13 bacterial-labeled cases.

PR-AUC and AUROC were summarized as mean ± standard deviation (SD) across test folds. We also pooled the held-out predictions, giving one prediction per patient, for curves, threshold metrics, and paired comparisons. The two summaries may rank models differently because pooled scores come from separately fitted models.

Uncertainty in pooled estimates was quantified with 95% confidence intervals (CIs) from 10,000 outcome-stratified patient bootstrap resamples, a number chosen to give stable interval limits. Each resample drew patients with replacement within each class, preserving the 105 bacterial and 530 nonbacterial patients, and the CI limits were the 2.5th and 97.5th percentiles. Paired differences were computed on the same resampled patients for both models. These intervals describe uncertainty in the observed out-of-fold predictions only; they do not capture variability from refitting or alternative data splits, nor fully account for dependence induced by overlapping training sets.

Two logistic-regression comparators used the same folds: a device-only model and a post hoc clinical-variable model using age, sex, and 20 symptom indicators. Preprocessing was fitted on training data only; the clinical-variable model additionally used training-derived class weights. Neither comparator received further tuning. Pairwise comparisons were exploratory and unadjusted for multiplicity; accordingly, intervals excluding zero were interpreted as exploratory signals of possible differences rather than confirmatory evidence that one model outperformed another. Separate comparator models do not estimate the incremental contribution of images to a combined clinical-plus-device model.

To assess whether image-model performance persisted within a single acquisition device, an exploratory post hoc sensitivity analysis trained and evaluated all five image models separately within the Samsung and Xiaomi strata. The analysis reused the original fold assignments after restricting the training, validation, and test sets to each device, retained the original training settings, and excluded 54 patients without identifiable EXIF device metadata. Within each device, pooled out-of-fold PR-AUC and AUROC, and paired differences relative to the original mixed-device models, were summarized using 10,000 outcome-stratified patient bootstrap resamples, with both models evaluated on the same resampled patients. Threshold-based measures were not summarized or interpreted in this analysis.

### 2.5. Computational Efficiency and Reproducibility

Efficiency measures were parameter count, multiply–accumulate operations (MACs), 32-bit floating-point (FP32) weight size, and median batch-1 latency at 224×224. Central processing unit (CPU) latency was measured on an Intel Xeon CPU @ 2.20 GHz (10 warm-up, 50 timed runs; default PyTorch 2.11.0 threading). NVIDIA A100 graphics processing unit (GPU) latency (30 warm-up, 100 timed runs) was reported for completeness but is not representative of resource-constrained devices. Patient-list and fold-assignment files were verified with SHA-256 hashes recorded in the public analysis code. Patient inclusion criteria, fixed folds, and training configuration are documented in Table S1.

## 3. Results

### 3.1. Primary Classification Performance

**Figure 4:**
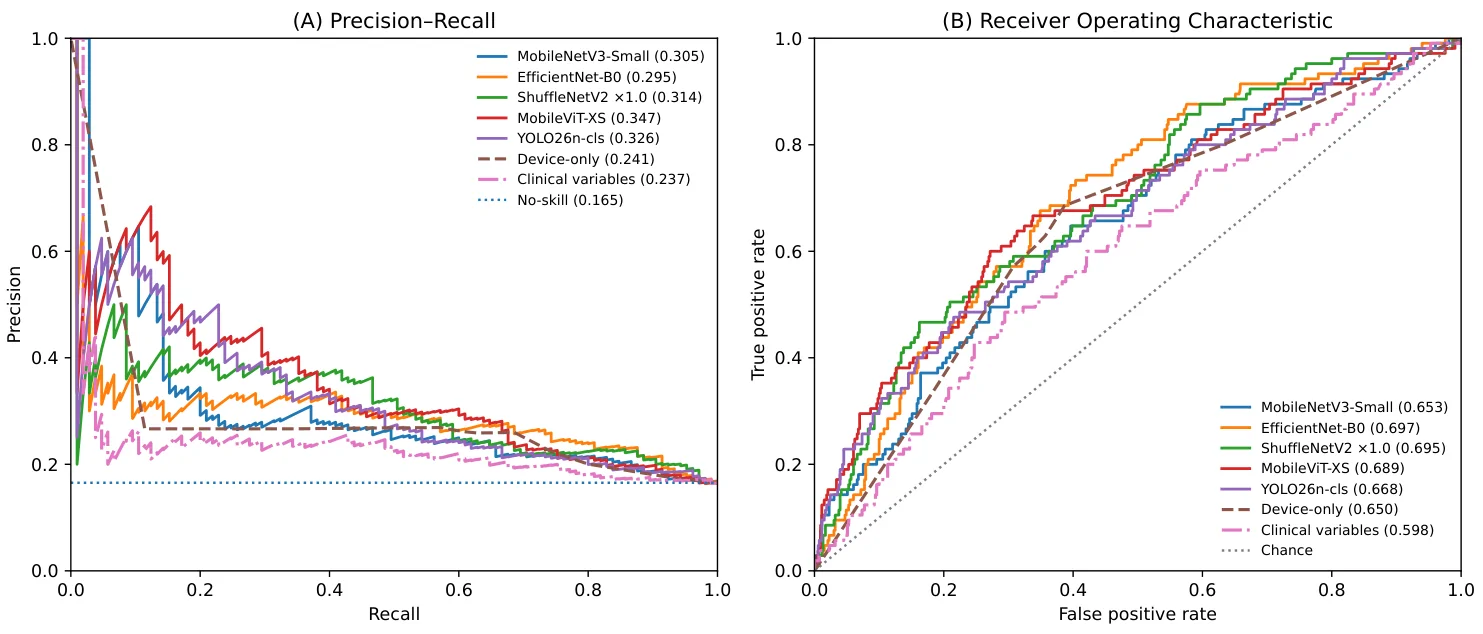
Pooled curves: (**A**) precision–recall and (**B**) receiver operating characteristic.

**Figure 5:**
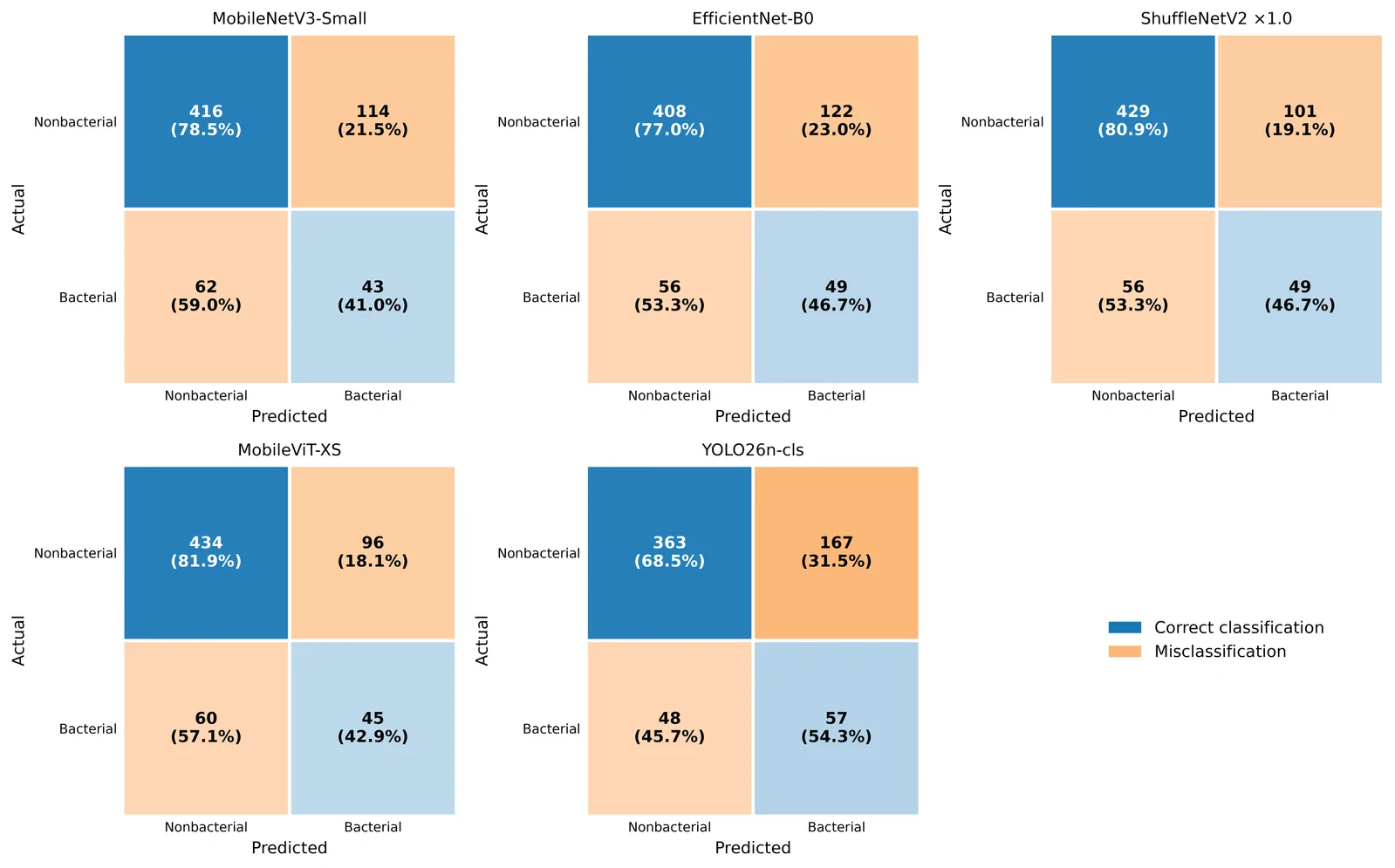
Pooled confusion matrices at threshold 0.50. Blue-shaded cells indicate correct classifications, whereas orange-shaded cells indicate misclassifications. Rows labeled “Actual” represent physician-consensus reference labels rather than microbiologically confirmed diagnoses.

Fold-mean PR-AUC ranged from 0.317 to 0.372 and AUROC from 0.663 to 0.708 (Table 5). Model ordering differed between fold-averaged and pooled estimates: ShuffleNetV2 had the highest fold-mean PR-AUC (0.372) and AUROC (0.708), MobileViT-XS had the highest pooled PR-AUC (0.347), and EfficientNet-B0 had the highest pooled AUROC (0.697). None of the 10 pairwise comparisons among image models had a 95% bootstrap confidence interval excluding zero for either primary metric (Table S2). Accordingly, no pairwise performance difference was statistically distinguishable from zero in this analysis, although this does not demonstrate equivalence. The pooled precision–recall and receiver operating characteristic curves are shown in Figure 4, and the corresponding confusion matrices at threshold 0.50 are shown in Figure 5.

The PR-AUC prevalence reference, equal to the bacterial-label prevalence, was 0.165; all pooled PR-AUC estimates and their 95% CIs exceeded this reference. Because the reference labels were physician judgments informed partly by the same throat images, the models may reproduce visual cues that contributed to those judgments, potentially making performance against this operational reference appear overly favorable. These estimates should therefore not be interpreted as diagnostic accuracy for microbiologically confirmed bacterial infection.

### 3.2. High-Sensitivity Operating Point

**Figure 6:**
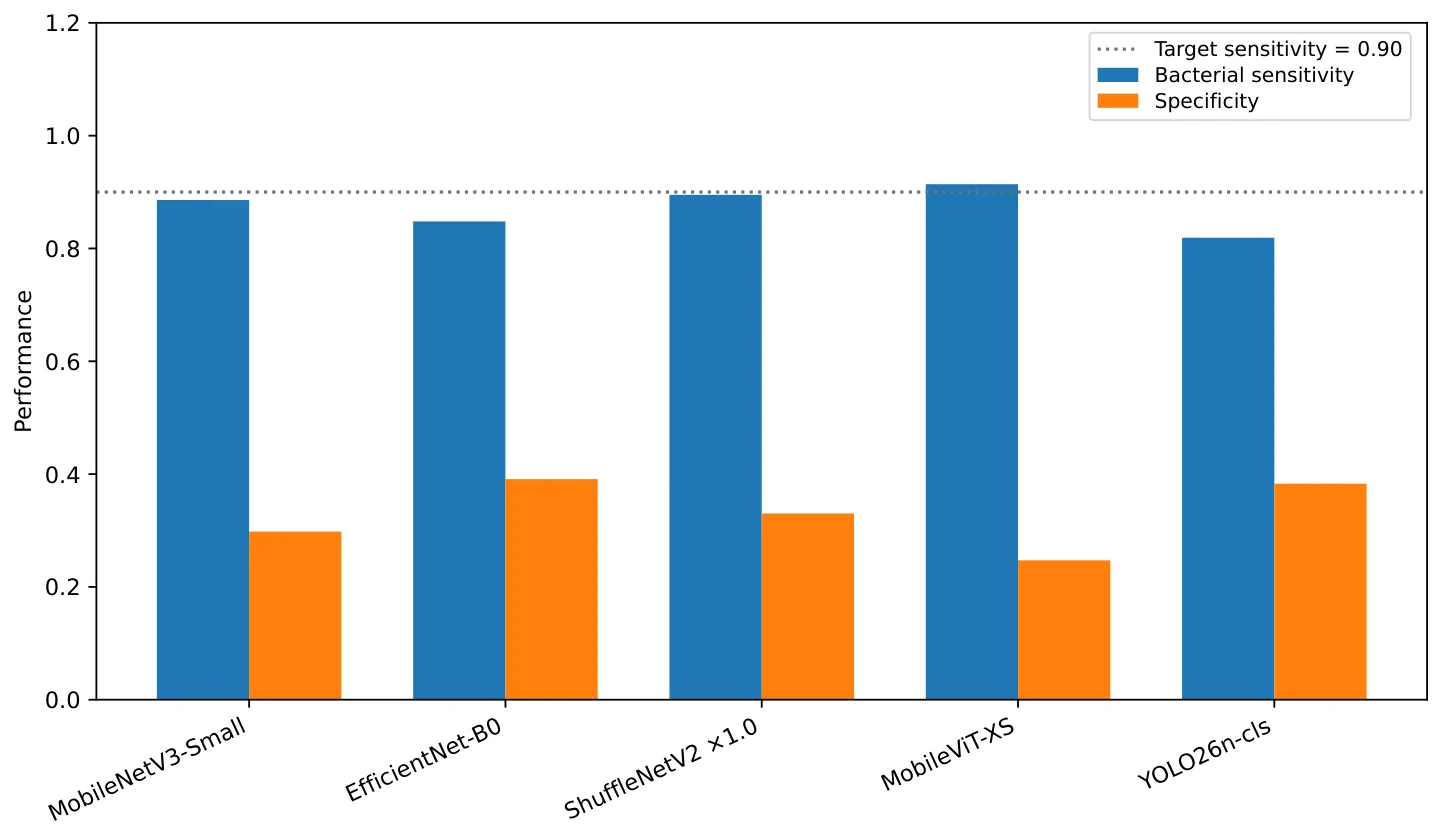
Sensitivity and specificity at validation-selected high-sensitivity thresholds.

Only MobileViT-XS achieved at least 90% sensitivity in pooled test predictions (0.91), with specificity of 0.25 (Table 6). Across models, positive predictive value (PPV) was 0.19–0.22, and 65–78 patients per 100 were flagged; thus, high sensitivity was reached only by flagging most patients, approximately four in five of whom had nonbacterial labels. The resulting sensitivity–specificity trade-offs are shown in Figure 6. These summaries combine five validation-selected thresholds; they neither show that every fold met the target nor define a single deployable cutoff. With only 12–13 bacterial-labeled cases per validation fold, the selected thresholds may be unstable and sensitive to individual predictions.

### 3.3. Non-Image Comparators and Device-Stratified Performance

The clinical-variable comparator achieved PR-AUC 0.237 and AUROC 0.598. Relative to this comparator, all five image models had higher PR-AUC and AUROC point estimates; unadjusted paired-difference confidence intervals excluded zero for both metrics with MobileViT-XS and for AUROC only with EfficientNet-B0 and ShuffleNetV2 (Table 7). At its high-sensitivity operating point, the clinical-variable model had sensitivity of 0.81, specificity of 0.21, and PPV of 0.17, each below the corresponding range for the image models (Table 6).

The device-only comparator achieved PR-AUC 0.241 and AUROC 0.650. Relative to this comparator, four image models had higher PR-AUC with unadjusted intervals excluding zero, but every AUROC difference interval included zero. The device-only and clinical-variable comparators were not clearly separated on either metric. Because the device-only model produced only 13 distinct scores, its PR-AUC estimate was coarse and was therefore interpreted alongside AUROC.

Bacterial-label prevalence differed by identified device: 9.2% for Samsung (33/358) and 26.9% for Xiaomi (60/223). Table 8 reports performance within each device stratum using models trained on the pooled, mixed-device dataset. Device-stratified PR-AUC was interpreted relative to each stratum’s prevalence reference. Within both strata, all models exceeded the prevalence reference for PR-AUC (Samsung 0.121–0.231; Xiaomi 0.322–0.437) and 0.500 for AUROC (Samsung 0.576–0.701; Xiaomi 0.552–0.659). The 95% CIs lay entirely above these references in 9 of 10 PR-AUC and 6 of 10 AUROC comparisons (Table S4). However, 9 of 10 within-device AUROCs were lower than the corresponding overall-cohort AUROCs (0.653–0.697), consistent with part of the overall discrimination reflecting between-device differences in label prevalence. In the EXIF-unavailable stratum (54 patients; 12 bacterial-labeled), all PR-AUCs exceeded the 0.222 prevalence reference (0.390–0.530) and all AUROCs exceeded 0.500 (0.536–0.669), as in the identified-device strata. These estimates are imprecise given the few bacterial-labeled cases (Table S3).

An exploratory post hoc analysis trained and evaluated each model separately within the Samsung and Xiaomi strata. All point estimates exceeded the device-specific prevalence reference for PR-AUC and 0.500 for AUROC, but only 6 of 10 PR-AUC and 5 of 10 AUROC confidence intervals lay entirely above these references (Table S4). Compared with the mixed-device-trained models in Table 8, point estimates were lower in 7 of 10 PR-AUC and 8 of 10 AUROC comparisons, but all paired-difference intervals except one included zero. Because device restriction also substantially reduced the training sample, the analysis cannot determine whether these differences reflect the removal of device-associated information or fewer training patients.

### 3.4. Computational Efficiency

**Figure 7:**
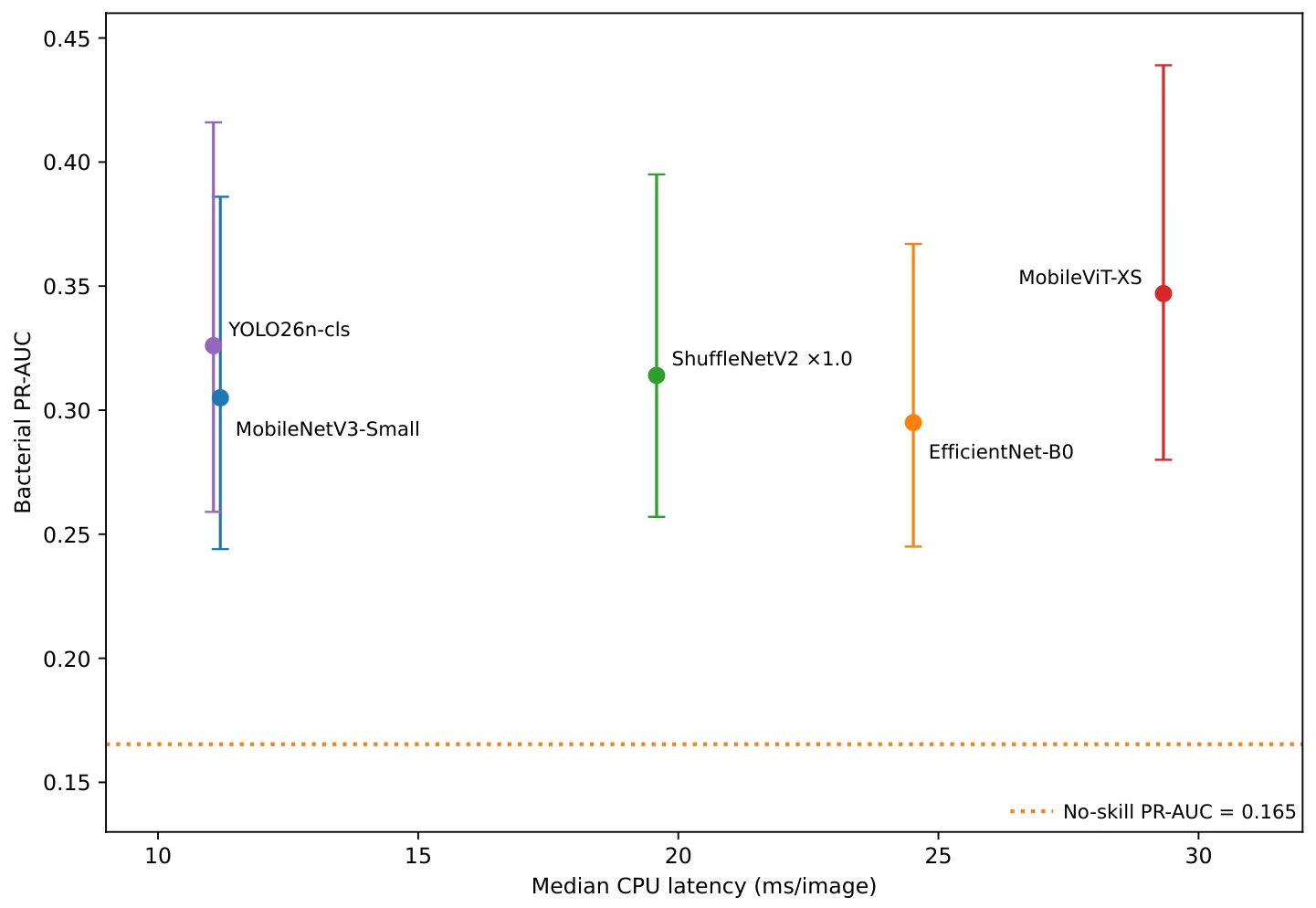
Bacterial PR-AUC versus median batch-1 CPU inference latency reported in Table 9. Vertical bars show 95% bootstrap CIs for pooled PR-AUC (Table 5). The dashed line marks the PR-AUC prevalence reference of 0.165.

Table 9 summarizes model size, computational cost, and inference latency. All five models had fewer than five million parameters (1.26–4.01 M). ShuffleNetV2 had the fewest parameters and the smallest FP32 weight size, and MobileNetV3-Small required the fewest MACs. YOLO26n-cls and MobileNetV3-Small had the lowest CPU latencies (11.07 and 11.20 ms), whereas MobileViT-XS required the most MACs and had the slowest CPU inference (29.33 ms). Figure 7 relates pooled PR-AUC to CPU latency. These timings were measured on a server CPU and do not represent performance on mobile or embedded devices.

## 4. Discussion

### 4.1. Principal Findings

The five models achieved fold-mean PR-AUCs of 0.317–0.372 and AUROCs of 0.663–0.708 for predicting physician-consensus labels. These metrics describe performance against those labels, not diagnostic accuracy for microbiologically confirmed bacterial infection or clinical utility. Among the five architectures, none was clearly superior: rankings changed between fold-averaged and pooled summaries, and no paired interval excluded zero. These intervals were conditional on the observed out-of-fold predictions and did not capture variability from refitting or alternative splits. When MobileNetV3-Small was retrained on one fold with three random seeds, PR-AUC varied with an SD of 0.062 (Panel C of Table S3), comparable to the 0.055 range in fold-mean PR-AUC across all five models. Although limited to one model and one fold, this suggests that small numerical differences between architectures should not be overinterpreted.

Acquisition-device identity was associated with the reference labels, and image-model AUROCs were not clearly separated from the device-only comparator (Table 7), so part of the image models’ performance may reflect acquisition- or cohort-associated information. However, within the Samsung and Xiaomi strata, 9 of 10 PR-AUC and 6 of 10 AUROC confidence intervals for the mixed-device-trained models lay entirely above their respective reference values (Table S4). Because device identity is constant within each stratum, this performance cannot be explained by device identity alone, although it may still reflect acquisition- or labeling-related factors rather than throat morphology. In the exploratory device-restricted analysis, performance estimates were generally lower but imprecise. Device restriction also reduced the training sample, so the observed changes could reflect either reduced device-associated information or fewer training patients. Device identity may capture differences in collection site, operator, lighting, other image-acquisition conditions, patient characteristics, and labeling practices; consequently, the device–label association should not be interpreted as a causal effect of the device itself. Similar acquisition effects have been documented in medical imaging [19,20].

All five image models had higher AUROCs than the clinical-variable comparator (0.598), with unadjusted paired-difference confidence intervals excluding zero for MobileViT-XS, EfficientNet-B0, and ShuffleNetV2; only MobileViT-XS also had a PR-AUC interval excluding zero (Table 7). Because incorporation bias may affect both the image models and the clinical-variable comparator, these exploratory differences do not establish that imaging adds diagnostic information beyond clinical variables. The symptom variables used by this comparator only partly followed established clinical scoring patterns. Cough was more common in the bacterial-labeled group (41.9% versus 29.1%), rhinorrhea was less common (19.0% versus 28.3%), and fever prevalence was similar (21.9% versus 21.7%). The cough pattern ran opposite to the Centor and McIsaac criteria, which award one point for absence of cough. Rhinorrhea is not included in either score, but its lower prevalence was directionally consistent with FeverPAIN’s combined absence-of-cough-or-coryza criterion [2]. These patterns concern physician-consensus labels and should not be interpreted as associations with confirmed GAS infection.

### 4.2. Previous Work and Clinical Interpretation

Our AUROC results were broadly consistent with Rahman’s analysis of the filtered PGUPharyngitis dataset, although protocol differences limit direct comparison [14,15]. At a threshold of 0.50, our models identified 41–54% of bacterial-labeled cases, whereas Rahman reported sensitivities of 10.4% and 8.3% for two image-only models without specifying the decision threshold. Rahman used focal loss and a consensus-score exclusion rule (640 patients; 15.0% bacterial-labeled). This study used inverse-frequency weighted cross-entropy and strict-majority labels, with additional exclusions for unmapped and duplicate images (635 patients; 16.5% bacterial-labeled). The architectures, training procedures, and model-selection criteria also differed. Because these factors were not compared experimentally, the sensitivity gap cannot be attributed to a single choice. Moreover, sensitivity depends on the applied threshold, so similar AUROCs can coexist with different sensitivities.

Increasing sensitivity required a substantial trade-off. Lower thresholds identified more bacterial-labeled cases but also flagged most patients, resulting in low positive predictive values. Thus, the ability to rank cases did not translate into a selective operating point at the evaluated high-sensitivity thresholds. These estimates should not be generalized directly to other clinical populations because positive predictive value depends on outcome prevalence and case mix.

The reference labels were also informed partly by the same throat images and overlapping clinical information, so both the image models and the clinical-variable comparator may partly reproduce the judgments used to establish those labels. Evaluations of other AI systems against expert decision-making, such as comparisons of ChatGPT 4.0’s biologic-therapy recommendations with rhinology-board decisions, likewise illustrate that agreement with clinicians is distinct from evidence of clinical benefit [27]. Overall, these findings support further investigation of throat imaging but do not establish its usefulness for antibiotic decisions or clinical triage. Future evaluations should therefore use a microbiological reference standard established independently of the evaluated inputs, such as throat culture or molecular testing. These evaluations should be conducted in external, prospectively collected cohorts and should assess calibration and clinically relevant decision thresholds.

### 4.3. Computational Efficiency and Future Evaluation

The models offered different computational advantages. ShuffleNetV2 had the smallest weight footprint, MobileNetV3-Small required the fewest MACs, and YOLO26n-cls and MobileNetV3-Small had the lowest CPU latencies. Because no pairwise difference in PR-AUC or AUROC between architectures was statistically distinguishable from zero, these efficiency differences may help prioritize architectures for further evaluation, provided that uncertainty in classification performance is considered.

The models’ compact size makes further evaluation on mobile hardware reasonable; however, the reported parameter counts, MACs, weight sizes, and server-side latencies indicate computational compactness rather than device-level deployability. Smart glasses are being explored in healthcare [28], although no wearable hardware was tested here. Any future application would require direct measurement of latency, memory use, and classification performance on the intended hardware under the intended acquisition conditions.

### 4.4. Limitations

Several limitations should guide interpretation of these findings. The analysis used a single public dataset with only 105 bacterial-labeled cases and lacked external validation; performance may therefore not generalize to other populations, devices, or acquisition settings. Because the labels reflected physician judgments informed by images and clinical information, they cannot establish bacterial etiology and may favor models using similar information. Excluding the 100 records with tied physician votes (13.5% of 742) removed cases for which the physicians did not reach a majority decision. This may have selected a cohort that was easier to classify according to the physician-consensus outcome, potentially yielding optimistic performance estimates relative to an unselected primary-care population. Performance on these cases could not be evaluated because they lacked a majority physician-consensus label. Discrepancies in the public release and missing site metadata further limited reproducibility and the interpretation of acquisition-related differences (Table S1).

Threshold stability was limited by the small number of bacterial-labeled cases in each validation fold. Pooled results also combine separately trained models and fold-specific thresholds rather than representing a single deployable model. Moreover, scores obtained through class-weighted training should not be interpreted as calibrated disease probabilities; calibration and clinical utility were not evaluated.

Uncertainty extends beyond the reported confidence intervals. Bootstrap intervals did not capture variability from retraining, and seed sensitivity was examined for only one architecture on one fold. Pairwise comparisons were unadjusted for multiple testing and should therefore be treated as exploratory. In addition, the preliminary learning-rate choice used the same source cohort, so the evaluation did not fully separate all development decisions from testing. Future studies should use a separate development cohort for hyperparameter selection and reserve an independent cohort for final evaluation.

The separate comparator analyses, including a post hoc clinical-variable model, did not establish whether images add value to a model already using clinical and device information. The exploratory device-restricted analysis excluded 54 patients without identifiable EXIF device metadata and relied on small device-specific samples, with only 33 Samsung and 60 Xiaomi bacterial-labeled cases overall and four and seven, respectively, per validation fold. These constraints limit precision and preclude causal attribution of the differences between device-restricted and mixed-device training. Formal sociodemographic subgroup and fairness analyses were not performed, given the limited demographic information and few bacterial-labeled cases available for precise estimates. Finally, efficiency measurements were obtained on server hardware and may not reflect mobile performance; peak inference memory was not measured.

## 5. Conclusions

Five lightweight models achieved fold-mean PR-AUCs of 0.317–0.372 and AUROCs of 0.663–0.708 when predicting physician-consensus bacterial versus nonbacterial labels from smartphone throat images, with no clearly superior architecture. These estimates describe classification performance against those labels rather than diagnostic accuracy for microbiologically confirmed bacterial infection. Acquisition device was associated with the reference labels, image-model AUROCs were not clearly distinguishable from that of the device-only comparator, and device-related confounding cannot be excluded. Some exploratory comparisons favored image models over the clinical-variable comparator, but neither comparator analysis establishes the incremental value of imaging. At validation-selected high-sensitivity thresholds, the models flagged 65–78% of patients with positive predictive values of only 19–22%, so clinical screening utility has not been established. All models had fewer than five million parameters, and the study provides a comparative benchmark of compact models; however, computational compactness alone does not demonstrate device-level or clinical deployability. Further investigation should prioritize standardized acquisition, device-balanced datasets, and validation in independent clinical cohorts using microbiological reference standards before any clinical application is considered.

## Figures and Tables

**Table 1:** Primary cohort characteristics.

Item	Value
Public spreadsheet records	742 patients
Mapped images in public release	741
Primary label definition	Strict majority of released physician votes
Final primary cohort	635 patients
Bacterial	105 (16.5%)
Nonbacterial	530 (83.5%)
Samsung	358 patients: 33 bacterial, 325 nonbacterial
Xiaomi	223 patients: 60 bacterial, 163 nonbacterial
EXIF device unavailable	54 patients: 12 bacterial, 42 nonbacterial
Available non-image clinical fields	Age, sex, and 20 binary symptom indicators

**Table 2:** Clinical characteristics of the final primary cohort by physician-consensus class.

Characteristic	Overall (*n* = 635)	Bacterial (*n* = 105)	Nonbacterial (*n* = 530)
Age, years, mean ± SD	28.53 ± 16.99	29.63 ± 16.75	28.31 ± 17.04
Female sex, *n* (%)	307 (48.3)	50 (47.6)	257 (48.5)
Sore throat, *n* (%)	290 (45.7)	57 (54.3)	233 (44.0)
Pyrexia (fever), *n* (%)	138 (21.7)	23 (21.9)	115 (21.7)
Cough, *n* (%)	198 (31.2)	44 (41.9)	154 (29.1)
Rhinorrhea, *n* (%)	170 (26.8)	20 (19.0)	150 (28.3)

**Table 3:** Lightweight architectures evaluated.

Model	Architecture Family	Parameters
ShuffleNetV2 ×1.0	Efficient convolutional neural network (CNN)	1,255,654
MobileNetV3-Small	Mobile CNN	1,519,906
YOLO26n-cls	Lightweight CNN classifier	1,533,666
MobileViT-XS	CNN–Transformer hybrid	1,933,618
EfficientNet-B0	Efficient scaled CNN	4,010,110

**Table 4:** Five-fold patient allocation; bacterial-labeled cases in parentheses.

Fold	Training, *n*	Validation, *n*	Held-Out Test, *n*
1	431 (71)	77 (12)	127 (22)
2	431 (71)	77 (12)	127 (22)
3	431 (71)	77 (13)	127 (21)
4	431 (72)	77 (13)	127 (20)
5	431 (72)	77 (13)	127 (20)

**Table 5:** Fold-averaged and pooled held-out performance against physician-consensus reference labels. Fold values are mean ± SD across five test folds; pooled PR-AUC and AUROC use one out-of-fold prediction per patient, with 95% bootstrap CIs. Model rankings differed between the two summaries. Sensitivity and specificity use pooled predictions at threshold 0.50.

Model	Fold PR-AUC	Pooled PR-AUC (95% CI)	Fold AUROC	Pooled AUROC (95% CI)	Sens.	Spec.
MobileNetV3-Small	0.328 ± 0.063	0.305 (0.244–0.386)	0.663 ± 0.041	0.653 (0.594–0.711)	0.410	0.785
EfficientNet-B0	0.317 ± 0.056	0.295 (0.245–0.367)	0.702 ± 0.028	0.697 (0.642–0.748)	0.467	0.770
ShuffleNetV2 ×1.0	0.372 ± 0.048	0.314 (0.257–0.395)	0.708 ± 0.031	0.695 (0.639–0.748)	0.467	0.809
MobileViT-XS	0.349 ± 0.074	0.347 (0.280–0.439)	0.697 ± 0.032	0.689 (0.630–0.745)	0.429	0.819
YOLO26n-cls	0.348 ± 0.021	0.326 (0.259–0.416)	0.673 ± 0.042	0.668 (0.608–0.725)	0.543	0.685

**Table 6:** Cross-validated performance at validation-selected high-sensitivity thresholds, evaluated against physician-consensus reference labels. PPV, positive predictive value; NPV, negative predictive value.

Model	Bacterial Sensitivity	Specificity	Patients Flagged per 100	PPV	NPV
MobileViT-XS	0.914	0.247	78.0	0.194	0.936
ShuffleNetV2 ×1.0	0.895	0.330	70.7	0.209	0.941
MobileNetV3-Small	0.886	0.298	73.2	0.200	0.929
EfficientNet-B0	0.848	0.391	64.9	0.216	0.928
YOLO26n-cls	0.819	0.383	65.0	0.208	0.914

**Table 7:** Paired differences versus non-image comparators (image model minus comparator; positive values favor the image model). The final row shows device-only minus clinical-variable. CIs are unadjusted 95% bootstrap intervals.

	Versus Device-Only		Versus Clinical-Variable	
Model	Δ PR-AUC (95% CI)	Δ AUROC (95% CI)	Δ PR-AUC (95% CI)	Δ AUROC (95% CI)
MobileViT-XS	+0.106 (0.033 to 0.194)	+0.039 (−0.021 to 0.102)	+0.111 (0.020 to 0.211)	+0.091 (0.011 to 0.172)
YOLO26n-cls	+0.084 (0.021 to 0.163)	+0.018 (−0.041 to 0.077)	+0.089 (−0.002 to 0.187)	+0.070 (−0.006 to 0.148)
ShuffleNetV2 ×1.0	+0.073 (0.005 to 0.154)	+0.045 (−0.015 to 0.106)	+0.077 (−0.008 to 0.170)	+0.097 (0.019 to 0.173)
MobileNetV3-Small	+0.064 (0.002 to 0.138)	+0.003 (−0.053 to 0.059)	+0.068 (−0.018 to 0.158)	+0.055 (−0.019 to 0.129)
EfficientNet-B0	+0.054 (−0.002 to 0.121)	+0.047 (−0.009 to 0.103)	+0.058 (−0.023 to 0.144)	+0.100 (0.024 to 0.175)
Device-only	—	—	+0.005 (−0.070 to 0.072)	+0.052 (−0.023 to 0.124)

**Table 8:** Performance of pooled-trained models evaluated within acquisition-device strata.

	Samsung: 33/358 Bacterial (9.2%)			Xiaomi: 60/223 Bacterial (26.9%)		
Model	PR-AUC	Δ PR	AUROC	PR-AUC	Δ PR	AUROC
Prevalence reference	0.092	0.000	0.500	0.269	0.000	0.500
EfficientNet-B0	0.208	+0.116	0.701	0.322	+0.053	0.552
MobileNetV3-Small	0.121	+0.029	0.576	0.393	+0.124	0.589
MobileViT-XS	0.134	+0.042	0.586	0.437	+0.168	0.659
ShuffleNetV2 ×1.0	0.231	+0.139	0.686	0.360	+0.091	0.606
YOLO26n-cls	0.146	+0.054	0.592	0.410	+0.141	0.620

Note: All models were trained using the pooled, mixed-device dataset. Δ PR denotes PR-AUC minus the device-specific bacterial-label prevalence; positive values indicate that the PR-AUC estimate exceeds that reference. Bootstrap confidence intervals and the device-restricted sensitivity analysis are reported in Table S4.

**Table 9:** Computational efficiency and median batch-1 inference latency at 224×224 input resolution. Weights denotes FP32 weight storage, not runtime memory.

Model	Params (M)	MACs (G)	Weights (MB)	CPU ms	CPU img/s	GPU ms
MobileNetV3-Small	1.52	0.061	5.93	11.20	89.3	6.59
EfficientNet-B0	4.01	0.414	15.58	24.52	40.8	9.86
ShuffleNetV2 ×1.0	1.26	0.152	4.96	19.58	51.1	8.11
MobileViT-XS	1.93	0.705	7.53	29.33	34.1	21.39
YOLO26n-cls	1.53	0.199	5.97	11.07	90.4	6.31

## Data Availability

The PGUPharyngitis dataset used in this study is publicly available on Figshare at DOI 10.6084/m9.figshare.28163513 under the Creative Commons Attribution 4.0 International (CC BY 4.0) license [14]. Representative images are reproduced in Figure 2 with attribution; the full image dataset and patient-level clinical records are not redistributed. Analysis code and non-identifying derived aggregate results are publicly available on Zenodo at DOI https://doi.org/10.5281/zenodo.23096431.

## Supplementary Materials

The following supporting information can be downloaded at https://www.mdpi.com/article/10.3390/diagnostics16213552/s1: Table S1, “Training configuration and reproducibility audit”; Table S2, “Paired comparisons among image models”; Table S3, “Additional fold-level and sensitivity analyses”; and Table S4, “Exploratory device-restricted sensitivity analysis”. A completed TRIPOD+AI checklist is provided as a separate submission file.

## Patient and Public Involvement Statement

No patients or members of the public were involved in the design or conduct of the present secondary analysis of publicly available data.
