# Supplementary material for: Comparing Lightweight Models for Physician-Consensus Pharyngitis Classification from Primary-Care Smartphone Images
Source: Diagnostics (Basel). 2026 Oct 28;16(21):3552. doi: 10.3390/diagnostics16213552 (PMC13761997; doi:10.3390/diagnostics16213552)
Supplement: Supplementary file 1 [file diagnostics-16-03552-s001.zip › supplementaryMaterials.pdf]

# Supplementary Materials

## Comparing Lightweight Models for Physician-Consensus Pharyngitis Classification from Primary-Care Smartphone Images

Adnan Miski*, Mazen A. Ismail, and Anas A. Makki

*Submitted to Diagnostics*
*Special Issue: AI for Precision Diagnostics: Enhancing Accuracy, Accessibility, and Outcomes*

* Correspondence: agmiski@kau.edu.sa

## Table S1. Training configuration and reproducibility audit

### Panel A. Final shared training protocol

| **Item** | **Setting** |
|---|---|
| Input | $224 \times 224$ RGB |
| Initialization | ImageNet-pretrained weights; all layers fine-tuned |
| Normalization | MobileNetV3-Small, EfficientNet-B0, and ShuffleNetV2: mean $(0.485, 0.456, 0.406)$ and SD $(0.229, 0.224, 0.225)$; MobileViT-XS and YOLO26n-cls: zero-to-one scaling without additional standardization |
| Optimizer | AdamW |
| Initial learning rate | $1 \times 10^{-4}$ |
| Weight decay | $1 \times 10^{-4}$ |
| Batch size | 32 |
| Maximum epochs | 60 |
| Loss | Inverse-frequency weighted cross-entropy; weights from training subset only |
| Learning-rate schedule | Cosine annealing to 1% of the initial learning rate |
| Early stopping | Validation weighted loss; patience 12 |
| Gradient clipping | 1.0 |
| Mixed precision | Enabled |
| Horizontal flip | $p = 0.5$ |
| Affine augmentation | Rotation ±7°; translation ±3%; scale 0.95–1.05 |
| Color jitter | Brightness 0.10; contrast 0.10; saturation 0.05; hue 0 |
| Cross-validation | Fixed five-fold patient-level split, stratified by class × device |
| Per-fold allocation | 431 training, 77 validation, 127 held-out test patients |
| Primary fold seeds | 10043, 10044, 10045, 10046, 10047 |
| Architecture-specific tuning | None |

**Panel B. Public-release audit and cohort reconstruction**

| Audit item | Observed result |
|---|---|
| Public spreadsheet records | 742 |
| Mapped readable throat images | 741 |
| Strict-majority labels before image/duplicate exclusions | 642 |
| Exact physician-vote ties excluded | 100 |
| Strict-majority case without mapped image | 1 |
| Byte-identical cross-patient image pairs | 3 pairs (6 records excluded; both records in each pair were removed because attribution of the duplicated image to either patient could not be established from the public release) |
| Final primary cohort | 635 patients: 105 bacterial, 530 nonbacterial |
| Reconstructed unanimous cases | 234 |
| Full-consensus cases reported in source article | 182 |
| Mean physician votes in released spreadsheet | 4.98 per patient |
| Acquisition strata in final cohort | Samsung SM-G998B: 358; Xiaomi 2201117SG: 223; EXIF unavailable: 54 |
| EXIF code 2201117SG | Manufacturer documentation identifies Redmi Note 11S; the source article describes Xiaomi Redmi 8 in one section and Redmi 8 Pro in another |

## Table S2. Paired comparisons among image models

Values are model A minus model B. Confidence intervals were obtained from 10,000 outcome-stratified patient bootstrap resamples using identical sampled patient indices for both models. These exploratory comparisons were not adjusted for multiplicity. Intervals summarize fixed out-of-fold predictions and do not include model-refitting or split variability.

| **Model A** | **Model B** | **Δ PR-AUC (95% CI)** | **Δ AUROC (95% CI)** |
|---|---|---|---|
| MobileNetV3-Small | EfficientNet-B0 | +0.010 (−0.057 to +0.076) | −0.044 (−0.096 to +0.008) |
| MobileNetV3-Small | ShuffleNetV2 ×1.0 | −0.009 (−0.086 to +0.068) | −0.042 (−0.104 to +0.020) |
| MobileNetV3-Small | MobileViT-XS | −0.042 (−0.122 to +0.030) | −0.036 (−0.092 to +0.019) |
| MobileNetV3-Small | YOLO26n-cls | −0.020 (−0.095 to +0.049) | −0.015 (−0.073 to +0.044) |
| EfficientNet-B0 | ShuffleNetV2 ×1.0 | −0.019 (−0.083 to +0.042) | +0.002 (−0.047 to +0.053) |
| EfficientNet-B0 | MobileViT-XS | −0.052 (−0.124 to +0.011) | +0.008 (−0.042 to +0.060) |
| EfficientNet-B0 | YOLO26n-cls | −0.031 (−0.101 to +0.033) | +0.029 (−0.025 to +0.084) |
| ShuffleNetV2 ×1.0 | MobileViT-XS | −0.033 (−0.107 to +0.036) | +0.006 (−0.053 to +0.064) |
| ShuffleNetV2 ×1.0 | YOLO26n-cls | −0.012 (−0.087 to +0.062) | +0.027 (−0.030 to +0.084) |
| MobileViT-XS | YOLO26n-cls | +0.022 (−0.060 to +0.103) | +0.021 (−0.038 to +0.080) |

# Table S3. Additional fold-level and sensitivity analyses

### Panel A. Full fold-by-fold image-model performance at threshold 0.50

| Model | Fold | n | B | PR-AUC | AUROC | Sens. | Spec. | Bact. F1 | Macro-F1 |
|---|---|---|---|---|---|---|---|---|---|
| MobileNetV3-Small | 1 | 127 | 22 | 0.428 | 0.698 | 0.500 | 0.790 | 0.400 | 0.617 |
| MobileNetV3-Small | 2 | 127 | 22 | 0.258 | 0.640 | 0.227 | 0.886 | 0.256 | 0.561 |
| MobileNetV3-Small | 3 | 127 | 21 | 0.303 | 0.602 | 0.286 | 0.802 | 0.250 | 0.538 |
| MobileNetV3-Small | 4 | 127 | 20 | 0.336 | 0.676 | 0.650 | 0.645 | 0.366 | 0.560 |
| MobileNetV3-Small | 5 | 127 | 20 | 0.313 | 0.697 | 0.400 | 0.804 | 0.327 | 0.583 |
| EfficientNet-B0 | 1 | 127 | 22 | 0.362 | 0.700 | 0.636 | 0.752 | 0.452 | 0.637 |
| EfficientNet-B0 | 2 | 127 | 22 | 0.274 | 0.660 | 0.273 | 0.848 | 0.273 | 0.560 |
| EfficientNet-B0 | 3 | 127 | 21 | 0.255 | 0.713 | 0.333 | 0.745 | 0.255 | 0.524 |
| EfficientNet-B0 | 4 | 127 | 20 | 0.309 | 0.698 | 0.550 | 0.766 | 0.393 | 0.611 |
| EfficientNet-B0 | 5 | 127 | 20 | 0.386 | 0.737 | 0.550 | 0.738 | 0.373 | 0.592 |
| ShuffleNetV2 ×1.0 | 1 | 127 | 22 | 0.339 | 0.665 | 0.500 | 0.771 | 0.386 | 0.604 |
| ShuffleNetV2 ×1.0 | 2 | 127 | 22 | 0.319 | 0.696 | 0.409 | 0.829 | 0.367 | 0.608 |
| ShuffleNetV2 ×1.0 | 3 | 127 | 21 | 0.369 | 0.712 | 0.476 | 0.830 | 0.408 | 0.633 |
| ShuffleNetV2 ×1.0 | 4 | 127 | 20 | 0.393 | 0.749 | 0.500 | 0.794 | 0.385 | 0.613 |
| ShuffleNetV2 ×1.0 | 5 | 127 | 20 | 0.441 | 0.719 | 0.450 | 0.822 | 0.375 | 0.615 |
| MobileViT-XS | 1 | 127 | 22 | 0.459 | 0.725 | 0.591 | 0.781 | 0.448 | 0.643 |
| MobileViT-XS | 2 | 127 | 22 | 0.337 | 0.674 | 0.273 | 0.924 | 0.333 | 0.612 |
| MobileViT-XS | 3 | 127 | 21 | 0.258 | 0.672 | 0.476 | 0.764 | 0.357 | 0.588 |
| MobileViT-XS | 4 | 127 | 20 | 0.372 | 0.737 | 0.350 | 0.841 | 0.318 | 0.588 |
| MobileViT-XS | 5 | 127 | 20 | 0.318 | 0.675 | 0.450 | 0.785 | 0.346 | 0.589 |
| YOLO26n-cls | 1 | 127 | 22 | 0.333 | 0.707 | 0.682 | 0.667 | 0.417 | 0.593 |
| YOLO26n-cls | 2 | 127 | 22 | 0.371 | 0.665 | 0.318 | 0.886 | 0.341 | 0.607 |
| YOLO26n-cls | 3 | 127 | 21 | 0.320 | 0.604 | 0.524 | 0.547 | 0.275 | 0.471 |
| YOLO26n-cls | 4 | 127 | 20 | 0.355 | 0.680 | 0.550 | 0.673 | 0.333 | 0.550 |
| YOLO26n-cls | 5 | 127 | 20 | 0.359 | 0.707 | 0.650 | 0.654 | 0.371 | 0.566 |

*Note:* B = bacterial cases in the held-out test fold. Balanced accuracy is omitted from the printed panel because it is determined by sensitivity and specificity; fold-level balanced accuracy can be calculated as (sensitivity + specificity)/2. Δ PR in Panel B is PR-AUC minus stratum prevalence.

### Panel A2. Mean ± SD across the five held-out folds

| Model | PR-AUC, mean ± SD | AUROC, mean ± SD |
|---|---|---|
| MobileNetV3-Small | 0.328 ± 0.063 | 0.663 ± 0.041 |
| EfficientNet-B0 | 0.317 ± 0.056 | 0.702 ± 0.028 |
| ShuffleNetV2 ×1.0 | 0.372 ± 0.048 | 0.708 ± 0.031 |
| MobileViT-XS | 0.349 ± 0.074 | 0.697 ± 0.032 |
| YOLO26n-cls | 0.348 ± 0.021 | 0.673 ± 0.042 |

*Note:* Fold-averaged and pooled estimates are complementary summaries. Because pooled probabilities come from separately trained fold models, numerical architecture ordering can differ between the two aggregation approaches.

**Panel B. EXIF-unavailable acquisition stratum**

| Model | n | B | Prevalence | PR-AUC | Δ PR | AUROC |
|---|---|---|---|---|---|---|
| MobileNetV3-Small | 54 | 12 | 0.222 | 0.417 | +0.195 | 0.635 |
| EfficientNet-B0 | 54 | 12 | 0.222 | 0.441 | +0.219 | 0.556 |
| ShuffleNetV2 ×1.0 | 54 | 12 | 0.222 | 0.394 | +0.172 | 0.641 |
| MobileViT-XS | 54 | 12 | 0.222 | 0.530 | +0.308 | 0.669 |
| YOLO26n-cls | 54 | 12 | 0.222 | 0.390 | +0.168 | 0.536 |

**Panel C. MobileNetV3-Small Fold-1 seed sensitivity**

| Seed | PR-AUC | AUROC | Best epoch |
|---|---|---|---|
| 10043 | 0.428 | 0.698 | 5 |
| 20043 | 0.304 | 0.653 | 5 |
| 30043 | 0.354 | 0.675 | 5 |
| Mean | 0.362 | 0.675 | 5 |
| SD | 0.062 | 0.023 | — |

*Note:* The seed analysis was limited to MobileNetV3-Small on Fold 1 and is descriptive rather than a general estimate of between-run variance.

## Table S4. Exploratory device-restricted sensitivity analysis

Models were trained and evaluated separately within the Samsung and Xiaomi strata (device-restricted) and compared with the original mixed-device models evaluated on the same patients (pooled-trained). Values are estimates with 95% CIs; differences are device-restricted minus pooled-trained.

### Panel A. PR-AUC estimates and paired differences

| Device | Model | Pooled-trained | Device-restricted | Difference |
|---|---|---|---|---|
| Samsung | EfficientNet-B0 | 0.208 (0.146–0.346) | 0.195 (0.119–0.324) | −0.013 (−0.133 to 0.084) |
| Samsung | MobileNetV3-Small | 0.121 (0.094–0.182) | 0.108 (0.088–0.163) | −0.013 (−0.057 to 0.027) |
| Samsung | MobileViT-XS | 0.134 (0.097–0.229) | 0.101 (0.086–0.136) | −0.032 (−0.114 to 0.006) |
| Samsung | ShuffleNetV2 ×1.0 | 0.231 (0.139–0.361) | 0.173 (0.127–0.279) | −0.057 (−0.167 to 0.057) |
| Samsung | YOLO26n-cls | 0.146 (0.101–0.264) | 0.191 (0.128–0.328) | +0.045 (−0.037 to 0.143) |
| Xiaomi | EfficientNet-B0 | 0.322 (0.263–0.416) | 0.324 (0.262–0.420) | +0.002 (−0.067 to 0.071) |
| Xiaomi | MobileNetV3-Small | 0.393 (0.309–0.502) | 0.344 (0.274–0.450) | −0.049 (−0.120 to 0.025) |
| Xiaomi | MobileViT-XS | 0.437 (0.354–0.558) | 0.387 (0.318–0.504) | −0.050 (−0.149 to 0.054) |
| Xiaomi | ShuffleNetV2 ×1.0 | 0.360 (0.295–0.465) | 0.376 (0.308–0.494) | +0.016 (−0.053 to 0.101) |
| Xiaomi | YOLO26n-cls | 0.410 (0.324–0.529) | 0.311 (0.264–0.406) | −0.099 (−0.188 to −0.015) |

### Panel B. AUROC estimates and paired differences

| Device | Model | Pooled-trained | Device-restricted | Difference |
|---|---|---|---|---|
| Samsung | EfficientNet-B0 | 0.701 (0.598–0.799) | 0.638 (0.537–0.735) | −0.063 (−0.151 to 0.030) |
| Samsung | MobileNetV3-Small | 0.576 (0.466–0.682) | 0.557 (0.460–0.657) | −0.019 (−0.100 to 0.070) |
| Samsung | MobileViT-XS | 0.586 (0.480–0.690) | 0.555 (0.461–0.648) | −0.031 (−0.137 to 0.073) |
| Samsung | ShuffleNetV2 ×1.0 | 0.686 (0.602–0.767) | 0.674 (0.574–0.765) | −0.012 (−0.108 to 0.084) |
| Samsung | YOLO26n-cls | 0.592 (0.491–0.692) | 0.642 (0.539–0.744) | +0.049 (−0.020 to 0.117) |
| Xiaomi | EfficientNet-B0 | 0.552 (0.467–0.635) | 0.551 (0.465–0.635) | −0.001 (−0.083 to 0.080) |
| Xiaomi | MobileNetV3-Small | 0.589 (0.503–0.675) | 0.554 (0.468–0.639) | −0.035 (−0.100 to 0.030) |
| Xiaomi | MobileViT-XS | 0.659 (0.573–0.740) | 0.640 (0.560–0.719) | −0.019 (−0.104 to 0.065) |
| Xiaomi | ShuffleNetV2 ×1.0 | 0.606 (0.521–0.690) | 0.612 (0.523–0.698) | +0.006 (−0.067 to 0.076) |
| Xiaomi | YOLO26n-cls | 0.620 (0.530–0.704) | 0.570 (0.488–0.651) | −0.050 (−0.125 to 0.025) |

*Notes:* PR-AUC prevalence references were 0.092 for Samsung and 0.269 for Xiaomi; the AUROC reference was 0.500. CIs were estimated using 10,000 outcome-stratified patient bootstrap resamples, and paired differences used identical resamples. Comparisons were exploratory and unadjusted for multiplicity; intervals including zero do not demonstrate equivalence. The 54 patients without identifiable EXIF device metadata were excluded. Samsung included 358 patients with 33 bacterial-labeled cases, and Xiaomi included 223 patients with 60 bacterial-labeled cases; each validation fold contained only 4 and 7 bacterial-labeled cases, respectively.
