# Supplementary material for: Comparing Lightweight Models for Physician-Consensus Pharyngitis Classification from Primary-Care Smartphone Images
Source: Diagnostics (Basel). 2026 Oct 28;16(21):3552. doi: 10.3390/diagnostics16213552 (PMC13761997; doi:10.3390/diagnostics16213552)
Supplement: Supplementary file 1 [file diagnostics-16-03552-s001.zip › TRIPOD_AI_Checklist.pdf]

Supplementary Materials

Comparing Lightweight Models for Physician-Consensus Pharyngitis Classification from Primary-Care Smartphone Images

Adnan Miski*, Mazen A. Ismail, and Anas A. Makki

Submitted to Diagnostics
Special Issue: AI for Precision Diagnostics: Enhancing Accuracy, Accessibility, and Outcomes

* Correspondence: agmiski@kau.edu.sa

Study scope. Secondary analysis evaluating five lightweight image-classification models using patient-level internal cross-validation. The predicted outcome is the physician-consensus bacterial versus nonbacterial label; it is not a microbiologically confirmed diagnosis.

Location convention. Locations refer to the current manuscript sections, tables, figures, and supplementary tables. Page numbers should be inserted after the final submission PDF is generated if requested by the journal.

Applicability. D = model development; E = model evaluation. Items marked "Not reported" identify information not stated in the current manuscript and do not imply that the activity occurred.

Part A. TRIPOD+AI for Abstracts

Item	Topic	Reported in	Reporting note
1	Title	Title; Abstract–Background/Objectives	Identifies the model evaluation, physician-consensus pharyngitis classification, primary-care setting, and smartphone images. The bacterial/nonbacterial outcome is explicit in the abstract.
2	Background	Abstract–Background/Objectives	States the potential primary-care assessment context and the need to evaluate performance and computational efficiency.
3	Objectives	Abstract–Background/Objectives	States that five lightweight models were compared for bacterial-versus-nonbacterial classification.
4	Data source	Abstract–Methods	Identifies the public PGUPharyngitis dataset.
5	Eligibility and setting	Abstract–Methods; Methods 2.1	Reports general-practice collection and the final cohort. Detailed reconstruction and exclusions are reported in Methods 2.1.
6	Outcome	Abstract–Background/Objectives and Methods	Defines bacterial versus nonbacterial physician-consensus labels and states the absence of microbiological confirmation.
7	Model type and evaluation	Abstract–Methods	Names all five architectures, pretrained initialization, fine-tuning, and patient-level five-fold cross-validation.
8	Performance measures	Abstract–Methods	Identifies PR-AUC and AUROC and states that computational efficiency was assessed.
9	Participants and outcome events	Abstract–Methods	Reports 635 patients, including 105 (16.5%) with bacterial consensus labels.
10	Predictors / inputs	Abstract–Methods	Identifies smartphone throat images and the device-only and clinical-variable non-image comparators.
11	Performance estimates and uncertainty	Abstract–Results; Table 5	The abstract reports fold-mean PR-AUC and AUROC ranges and threshold summaries, but does not include confidence intervals. Model-level pooled 95% CIs are reported in Table 5.

Item	Topic	Reported in	Reporting note
12	Interpretation	Abstract–Conclusions	Provides cautious interpretation, states that device-related confounding cannot be excluded, does not claim clinical readiness, and calls for validation in independent clinical cohorts.
13	Registration	**Not reported**	No study-registration identifier is reported in the current manuscript.

Part B. Full TRIPOD+AI Checklist

Item	D/E	Topic	Reported in	Reporting note
1	D;E	Title	Title; Abstract	Identifies the comparative model evaluation, target setting, image source, and physician-consensus pharyngitis outcome; the bacterial/nonbacterial distinction is stated in the abstract.
2	D;E	Abstract	Abstract	Structured abstract reports background/objectives, methods, results, and conclusions; see Part A.
3a	D;E	Healthcare context and rationale	Introduction	Describes the primary-care context, antibiotic-stewardship motivation, prior image-based work, computational-efficiency question, and acquisition-device concerns.
3b	D;E	Target population and intended purpose	Introduction, final two paragraphs	Frames the target population as patients presenting with pharyngitis/common-cold-related symptoms and the possible role as future decision support rather than a replacement for microbiological testing.
3c	D;E	Known health inequalities	**Not reported**	No specific pre-existing health inequalities across sociodemographic groups are described. The absence of formal fairness analysis is acknowledged in the Limitations.
4	D;E	Objectives	Introduction, final paragraph	States the comparative evaluation objective, performance and efficiency outcomes, non-image comparators, and intended scope.
5a	D;E	Data sources and representativeness	Methods 2.1	Identifies the public PGUPharyngitis dataset, collection through general practitioners, two Iranian regions, and use as a secondary-analysis cohort.
5b	D;E	Participant-data dates	Methods 2.1	Reports October 2023 to May 2024.
6a	D;E	Study setting	Methods 2.1	Reports general-practice collection in two regions of Iran.
6b	D;E	Eligibility criteria	Methods 2.1; Figure 1; Table 1	Reports the source quality review, vote-based cohort reconstruction, and all exclusions used in this analysis. Detailed original recruitment criteria remain available through the cited source study.
6c	D;E	Treatments	**Not applicable**	No treatment intervention or treatment-effect prediction was evaluated.
7	D;E	Data preparation and quality checks	Methods 2.1–2.2; Supplementary Table S1	Reports vote reconstruction, image mapping and readability, duplicate checks, RGB input in Supplementary Table S1, resizing, augmentation, scaling, and normalization.
8a	D;E	Outcome definition	Abstract–Methods; Methods 2.1; Results 3.1; Discussion 4.2 and 4.4	Defines the strict-majority physician-consensus outcome, positive class, source nonbacterial category, absence of microbiological confirmation, and potential incorporation bias. No prognostic time horizon applies.
8b	D;E	Outcome-assessor characteristics	Methods 2.1; cited source study	Reports that released physician assessments were used and that one physician examined the throat directly while others reviewed images; fuller assessor details are deferred to the source study.
8c	D;E	Blinding of outcome assessment	**Not reported**	The current manuscript does not report blinding of the physicians who produced the source labels.
9a	D	Choice of predictors / inputs	Methods 2.2 and 2.4	Primary models use throat images; comparators use acquisition device alone or age, sex, and 20 symptom indicators.
9b	D;E	Predictor definition and handling	Methods 2.1–2.4; Table 1	Defines the image input, acquisition-device strata, and clinical-variable set and reports image preprocessing. Detailed source measurement timing and variable-level missingness are not reported.
9c	D;E	Subjective predictor assessors	Cited source study; **Not reported** in current manuscript	Source symptoms were reused as released; the current manuscript does not provide qualifications or demographics of the original symptom assessors.

Item	D/E	Topic	Reported in	Reporting note
10	D;E	Study size	Methods 2.1; Table 1; Limitations	Study size comprised all records meeting the reconstruction and image-quality criteria (635 patients; 105 bacterial labels). No formal prospective sample-size calculation was performed; the limited positive-class size is acknowledged.
11	D;E	Missing data and exclusions	Methods 2.1; Figure 1; Table 1; Supplementary Table S1	Reports vote ties, the unmapped image, duplicate-image exclusions, and the EXIF-unavailable stratum. Variable-level missingness for age, sex, and symptom indicators is not described.
12a	D	Use and partitioning of data	Methods 2.3; Table 4; Figure 3	Describes fixed patient-level five-fold training, validation, and held-out testing, with every patient tested once and development decisions separated from final scoring.

Item	D/E	Topic	Reported in	Reporting note
12b	D	Handling and transformation of inputs	Methods 2.2 and 2.4; Supplementary Table S1	Reports 224×224 RGB input, training-only augmentation, model-specific normalization or scaling, and training-only fitting of comparator preprocessing.
12c	D	Model type and development procedure	Methods 2.2–2.3; Supplementary Table S1	Specifies architecture families, ImageNet initialization, end-to-end fine-tuning, optimizer, learning rate, loss, class weighting, schedule, early stopping, folds, seeds, and lack of architecture-specific tuning.
12d	D;E	Heterogeneity across clusters	Methods 2.4; Results 3.3; Supplementary Table S4; Limitations	No formal centre-level heterogeneity analysis was possible because site metadata were unavailable. Acquisition-device-stratified performance is reported descriptively, and an exploratory post hoc analysis trained and evaluated all models separately within the Samsung and Xiaomi strata.
12e	D;E	Performance measures and comparisons	Methods 2.4	Specifies PR-AUC, AUROC, threshold metrics, fold summaries, pooled estimates, validation-selected screening thresholds, bootstrap CIs, paired comparisons, the exploratory multiplicity interpretation, and the post hoc device-restricted sensitivity analysis.
12f	E	Model updating after evaluation	**Not applicable**	No recalibration or other post-evaluation model updating was performed.
12g	E	Generation of evaluation predictions	Methods 2.3–2.4	Held-out predictions were produced by fold-specific selected checkpoints; validation-selected thresholds were applied unchanged to the corresponding test folds.
13	D;E	Class imbalance	Methods 2.3–2.4	Uses inverse-frequency weighted cross-entropy and emphasizes PR-AUC for the minority bacterial class. No post hoc probability recalibration was performed.
14	D;E	Fairness methods	Discussion 4.4	States that formal sociodemographic subgroup and fairness analyses were not performed because demographic information was limited and the bacterial sample was small.
15	D	Model output and thresholds	Methods 2.4	Evaluates probability scores at 0.50 and exploratory validation-selected thresholds targeting at least 90% bacterial sensitivity.
16	D;E	Development/evaluation data differences	Methods 2.3	All folds derive from the same cleaned cohort; training, validation, and held-out roles and their separation are reported.
17	D;E	Ethics and consent	Methods 2.1; Institutional Review Board Statement; Informed Consent Statement	Reports source ethics approval, original written informed consent, anonymized public-data reuse, and absence of new recruitment or data collection.
18a	D;E	Funding	Funding; Acknowledgments	Identifies King Abdulaziz University Endowment (WAQF) funding and acknowledges WAQF and the Deanship of Scientific Research. The funder's role is not otherwise described.
18b	D;E	Conflicts of interest	Conflicts of Interest	Declares no conflict of interest.
18c	D;E	Protocol availability	Methods 2.1–2.5; Supplementary Table S1	No standalone protocol document is identified. The cohort definition, fixed folds, shared training protocol, statistical analysis, and reproducibility audit are documented in the manuscript and Supplementary Table S1.
18d	D;E	Study registration	**Not reported**	No study-registration identifier or explicit registration statement is provided.
18e	D;E	Data sharing	Data Availability Statement	Provides the public Figshare DOI and CC BY 4.0 license for PGUPharyngitis and explains that the full dataset is not redistributed.
18f	D;E	Code sharing	Data Availability Statement	Provides a Zenodo DOI for analysis code and non-identifying derived aggregate results.
19	D;E	Patient and public involvement	Patient and Public Involvement Statement	States that no patients or members of the public were involved in the design or conduct of this secondary analysis.
20a	D;E	Participant flow	Methods 2.1; Figure 1; Table 1	Reports source records, exclusions, final cohort, and physician-consensus class counts.
20b	D;E	Participant characteristics	Tables 1–2	Reports class and acquisition-device distributions and age, sex, and selected symptom distributions by physician-consensus class.
20c	E	Development/evaluation distributions	Methods 2.3; Table 4	Reports per-fold sample and bacterial-case counts and joint stratification by class and device. Broader predictor distributions by fold are not shown.
21	D;E	Participants and events in analyses	Table 4; Results	Reports training, validation, and held-out counts and bacterial events for all five folds; every patient contributes one held-out prediction per model.
22	D	Full model specification and access	Methods 2.2–2.3; Supplementary Table S1; Data Availability Statement	Reports architectures, parameter counts, training settings, seeds, and a code DOI. Availability of trained model weights is not stated.

Item	D/E	Topic	Reported in	Reporting note
23a	D;E	Performance and uncertainty	Results 3.1–3.4; Tables 5–8; Supplementary Tables S2–S4	Reports fold means and SDs, pooled 95% CIs, threshold metrics, paired differences, acquisition-device strata with bootstrap CIs, the device-restricted sensitivity analysis, and the limited seed-sensitivity analysis. Formal sociodemographic subgroup performance was not assessed.
23b	D;E	Heterogeneity across clusters	Results 3.3; Supplementary Tables S3–S4; Limitations	Reports device-stratified exploratory results, including the EXIF-unavailable stratum, and device-restricted training and evaluation within the Samsung and Xiaomi strata. Formal site/centre heterogeneity was not evaluated.
24	E	Model updating results	**Not applicable**	No model updating was performed.
25	D;E	Interpretation and fairness	Discussion 4.1–4.4	Interprets performance, uncertainty, device association, comparison with prior work, clinical limitations, efficiency, and the absence of formal fairness analysis.
26	D;E	Limitations	Discussion 4.4	Discusses cohort size, physician-consensus reference labels, incorporation bias, potentially optimistic estimates from tied-case exclusion, internal validation, threshold fragility, model-refitting uncertainty, seed sensitivity, multiplicity, separate comparators, the imprecision of the device-restricted analysis, fairness, server timing, unmeasured peak inference memory, and missing site metadata.
27a	D	Poor or unavailable inputs in implementation	**Not applicable** at current study stage; Discussion 4.3–4.4	The work does not claim an implementation-ready system and does not define a future image-quality rejection procedure.
27b	D	User interaction and required expertise	**Not applicable** at current study stage; Discussion 4.3	No clinical, portable-device, or wearable workflow was implemented or tested.
27c	D;E	Future research and generalizability	Introduction; Discussion 4.2–4.4; Conclusions	Calls for independent microbiological reference standards in external, prospectively collected cohorts with assessment of calibration and clinically relevant thresholds; separate development and evaluation cohorts; standardized acquisition; device-balanced datasets; and device-level measurement of latency and memory before use on other camera-equipped platforms.

Reference

Collins GS, Moons KGM, Dhiman P, et al. TRIPOD+AI statement: updated guidance for reporting clinical prediction models that use regression or machine learning methods. *BMJ*. 2024;385:e078378. doi:10.1136/bmj-2023-078378.
